# Monocrotophos: dimethyl (*E*)-1-methyl-2-(methyl­carbamo­yl)ethenyl phosphate

**DOI:** 10.1107/S1600536811003898

**Published:** 2011-02-09

**Authors:** Sanghun Cheon, Tae Ho Kim, Ki-Min Park, Jineun Kim

**Affiliations:** a Department of Chemistry and Research Institute of Natural Sciences, Gyeongsang National University, Jinju 660-701, Republic of Korea

## Abstract

In the title compound, C_7_H_14_NO_5_P, the phosphate group displays rotational disorder of three O atoms with an occupancy ratio of 0.832 (6):0.167 (6). The dihedral angle between the acryl­amide group and PO_2_ plane of the phosphate group is 75.69 (7)°. In the crystal, inter­molecular N—H⋯O and C—H⋯O hydrogen bonds link the molecules.

## Related literature

For the toxicity and insecticidal properties of the title compound, see: Dureja (1989 ▶); Chakravarthi *et al.* (2007 ▶). For related structures, see: Osman & El-Samahy (2007 ▶).
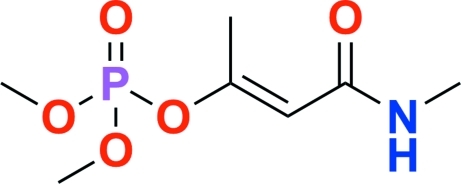


## Experimental

### 

#### Crystal data

C_7_H_14_NO_5_P
*M* *_r_* = 223.16
Monoclinic, 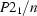

*a* = 10.0498 (2) Å
*b* = 11.3501 (2) Å
*c* = 10.4587 (2) Å
β = 115.377 (1)°
*V* = 1077.87 (4) Å^3^
*Z* = 4
Mo *K*α radiation
μ = 0.25 mm^−1^
*T* = 173 K
0.35 × 0.35 × 0.25 mm

#### Data collection

Bruker APEXII CCD diffractometer
Absorption correction: multi-scan (*SADABS*; Sheldrick, 1996 ▶) *T*_min_ = 0.917, *T*_max_ = 0.940
17626 measured reflections
2673 independent reflections
2411 reflections with *I* > 2σ(*I*)
*R*_int_ = 0.032

#### Refinement

*R*[*F* ^2^ > 2σ(*F* ^2^)] = 0.040
*wR*(*F* ^2^) = 0.119
*S* = 1.08
2673 reflections
155 parameters
H-atom parameters constrained
Δρ_max_ = 0.37 e Å^−3^
Δρ_min_ = −0.38 e Å^−3^

### 

Data collection: *APEX2* (Bruker, 2006 ▶); cell refinement: *SAINT* (Bruker, 2006 ▶); data reduction: *SAINT*; program(s) used to solve structure: *SHELXTL* (Sheldrick, 2008 ▶); program(s) used to refine structure: *SHELXTL*; molecular graphics: *SHELXTL* and *DIAMOND* (Brandenburg, 1998 ▶); software used to prepare material for publication: *SHELXTL*.

## Supplementary Material

Crystal structure: contains datablocks global, I. DOI: 10.1107/S1600536811003898/jh2262sup1.cif

Structure factors: contains datablocks I. DOI: 10.1107/S1600536811003898/jh2262Isup2.hkl

Additional supplementary materials:  crystallographic information; 3D view; checkCIF report

## Figures and Tables

**Table 1:** Hydrogen-bond geometry (Å, °)

*D*—H⋯*A*	*D*—H	H⋯*A*	*D*⋯*A*	*D*—H⋯*A*
N1—H1*N*⋯O1^i^	0.88	2.03	2.902 (2)	169
C4—H4*B*⋯O2^ii^	0.98	2.43	3.319 (2)	151

Symmetry codes: (i) 
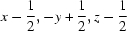
; (ii) 
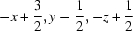
.

**Fig. 1.**
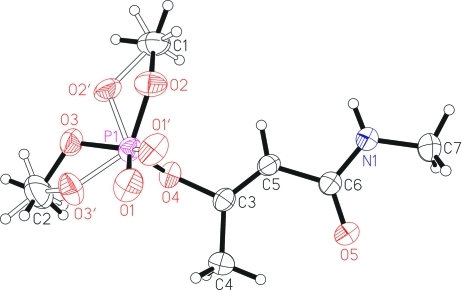
The molecular structure of the title compound with the atom numbering scheme: the major part is drawn with solid lines, the minor one with open lines. Displacement ellipsoids are drawn at the 50% probability level. H atoms are presented as a small spheres of arbitrary radii.

**Fig. 2.**
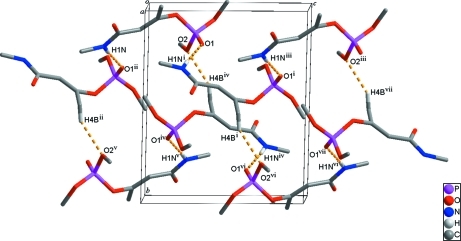
Crystal packing of the title compound with intermolecular N—H···O and C—H···O interactions shown as dashed lines. H atoms not involved in intermolecular interactions have been omitted for clarity. [Symmetry codes: (i) x + 1/2, -y + 1/2, z + 1/2; (ii) x - 1/2, -y + 1/2, z - 1/2; (iii) x + 1, y, z + 1; (iv) -x + 1.5, y + 1/2, -z + 1/2; (v) -x + 1, -y + 1, -z; (vi) -x + 2, -y + 1, -z + 1; (vii) -x + 2.5, y + 1/2, -z + 1.5.)

## supplementary crystallographic information

### Comment

Monocrotophos (systematic name: dimethyl (*E*)-1-methyl-2- (methylcarbamoyl)vinyl phosphate), is a kind of insecticide with a wide range of insects and mites (Dureja, 1989; Chakravarthi *et al.*, 2007). However it's crystal structure has not been reported yet.

In the title compound (Scheme 1, Fig.1), the phosphate group displays rotational disorder with occupancies of 0.832 (6):0.167 (6). The dihedral angle between the acrylamide group and PO_2_ planes (P1/O1/O2) of the phosphate group is 75.69 (7)°. All bond lengths and bond angles are normal and comparable to those observed in similar structures (Osman & El-Samahy, 2007).

In the crystal structure, as shown in Fig. 2, weak intermolecular N—H···O and C—H···O hydrogen bonds are observed (Table 1). These intermolecular interactions may be contribute to the stabilization of the packing.

### Experimental

The title compound was purchased from the Dr. Ehrenstorfer GmbH Company. Slow evaporation of a solution in CH_2_Cl_2_ gave single crystals suitable for X-ray analysis.

### Refinement

During refinement, atoms O1, O2 and O3 of the phosphate group are disordered and were refined using a split model. The corresponding site-occupation factors were refined so that their sum was unity [0.832 (6) and 0.167 (6)]. All H-atoms were positioned geometrically and refined using a riding model with d(N—H) = 0.88 Å, *U*_iso_ = 1.2*U*_eq_(N) for NH, d(C—H) = 0.98 Å, *U*_iso_ = 1.2*U*_eq_(C) for CH and d(C—H) = 0.98 Å, *U*_iso_ = 1.5*U*_eq_(C) for CH_3_ groups.

### Crystal data

**Table:**

C_7_H_14_NO_5_P	*F*(000) = 472
*M**_r_* = 223.16	*D*_x_ = 1.375 Mg m^−^^3^
Monoclinic, *P*2_1_/*n*	Mo *K*α radiation, λ = 0.71073 Å
Hall symbol: -P 2yn	Cell parameters from 9925 reflections
*a* = 10.0498 (2) Å	θ = 2.4–28.3°
*b* = 11.3501 (2) Å	µ = 0.25 mm^−^^1^
*c* = 10.4587 (2) Å	*T* = 173 K
β = 115.377 (1)°	Block, colourless
*V* = 1077.87 (4) Å^3^	0.35 × 0.35 × 0.25 mm
*Z* = 4	

### Data collection

**Table:**

Bruker APEXII CCD diffractometer	2673 independent reflections
Radiation source: fine-focus sealed tube	2411 reflections with *I* > 2σ(*I*)
graphite	*R*_int_ = 0.032
φ and ω scans	θ_max_ = 28.3°, θ_min_ = 2.4°
Absorption correction: multi-scan (*SADABS*; Sheldrick, 1996)	*h* = −13→13
*T*_min_ = 0.917, *T*_max_ = 0.940	*k* = −15→15
17626 measured reflections	*l* = −13→13

### Refinement

**Table:**

Refinement on *F*^2^	Primary atom site location: structure-invariant direct methods
Least-squares matrix: full	Secondary atom site location: difference Fourier map
*R*[*F*^2^ > 2σ(*F*^2^)] = 0.040	Hydrogen site location: inferred from neighbouring sites
*wR*(*F*^2^) = 0.119	H-atom parameters constrained
*S* = 1.08	*w* = 1/[σ^2^(*F*_o_^2^) + (0.0647*P*)^2^ + 0.3928*P*] where *P* = (*F*_o_^2^ + 2*F*_c_^2^)/3
2673 reflections	(Δ/σ)_max_ < 0.001
155 parameters	Δρ_max_ = 0.37 e Å^−^^3^
0 restraints	Δρ_min_ = −0.38 e Å^−^^3^

### Special details

**Table:**

Geometry. All e.s.d.'s (except the e.s.d. in the dihedral angle between two l.s. planes) are estimated using the full covariance matrix. The cell e.s.d.'s are taken into account individually in the estimation of e.s.d.'s in distances, angles and torsion angles; correlations between e.s.d.'s in cell parameters are only used when they are defined by crystal symmetry. An approximate (isotropic) treatment of cell e.s.d.'s is used for estimating e.s.d.'s involving l.s. planes.
Refinement. Refinement of *F*^2^ against ALL reflections. The weighted *R*-factor *wR* and goodness of fit *S* are based on *F*^2^, conventional *R*-factors *R* are based on *F*, with *F* set to zero for negative *F*^2^. The threshold expression of *F*^2^ > σ(*F*^2^) is used only for calculating *R*-factors(gt) *etc*. and is not relevant to the choice of reflections for refinement. *R*-factors based on *F*^2^ are statistically about twice as large as those based on *F*, and *R*- factors based on ALL data will be even larger.

### Fractional atomic coordinates and isotropic or equivalent isotropic displacement parameters (Å^2^)

**Table:**

	*x*	*y*	*z*	*U*_iso_*/*U*_eq_	Occ. (<1)
P1	0.72597 (4)	0.12670 (3)	0.30753 (4)	0.02990 (14)	
O1	0.87089 (17)	0.17794 (17)	0.35222 (16)	0.0442 (4)	0.833 (2)
O2	0.60357 (16)	0.22189 (12)	0.25429 (17)	0.0421 (4)	0.833 (2)
O3	0.69477 (16)	0.05711 (13)	0.41970 (14)	0.0404 (4)	0.833 (2)
O1'	0.8042 (9)	0.2366 (7)	0.2975 (8)	0.0385 (17)	0.167 (2)
O2'	0.5877 (7)	0.1471 (7)	0.3323 (7)	0.0414 (18)	0.167 (2)
O3'	0.8319 (8)	0.0599 (7)	0.4465 (7)	0.0446 (19)	0.167 (2)
O4	0.68546 (12)	0.03323 (9)	0.18490 (11)	0.0319 (2)	
O5	0.75176 (15)	0.08602 (13)	−0.19560 (13)	0.0486 (3)	
N1	0.55391 (16)	0.20368 (14)	−0.26018 (15)	0.0423 (3)	
H1N	0.4883	0.2357	−0.2360	0.051*	
C1	0.44938 (19)	0.19657 (18)	0.2069 (2)	0.0494 (4)	
H1A	0.3929	0.2700	0.1787	0.074*	0.833 (2)
H1B	0.4335	0.1597	0.2840	0.074*	0.833 (2)
H1C	0.4168	0.1428	0.1259	0.074*	0.833 (2)
H1D	0.3692	0.2054	0.2359	0.074*	0.167 (2)
H1E	0.4189	0.1421	0.1267	0.074*	0.167 (2)
H1F	0.4726	0.2735	0.1790	0.074*	0.167 (2)
C2	0.7899 (3)	−0.0384 (2)	0.4980 (2)	0.0655 (6)	
H2A	0.7542	−0.0715	0.5641	0.098*	0.833 (2)
H2B	0.8904	−0.0087	0.5510	0.098*	0.833 (2)
H2C	0.7898	−0.0998	0.4321	0.098*	0.833 (2)
H2D	0.8730	−0.0660	0.5840	0.098*	0.167 (2)
H2E	0.7597	−0.1009	0.4265	0.098*	0.167 (2)
H2F	0.7073	−0.0181	0.5201	0.098*	0.167 (2)
C3	0.72637 (16)	0.04930 (12)	0.07257 (14)	0.0284 (3)	
C4	0.86253 (19)	−0.01539 (16)	0.09550 (19)	0.0442 (4)	
H4A	0.8863	−0.0020	0.0151	0.066*	
H4B	0.8477	−0.0998	0.1041	0.066*	
H4C	0.9438	0.0128	0.1825	0.066*	
C5	0.63716 (16)	0.11200 (13)	−0.03706 (15)	0.0298 (3)	
H5A	0.5546	0.1478	−0.0310	0.036*	
C6	0.65555 (17)	0.13105 (13)	−0.16923 (16)	0.0324 (3)	
C7	0.5494 (3)	0.2307 (3)	−0.3968 (2)	0.0675 (7)	
H7A	0.4682	0.2854	−0.4472	0.101*	
H7B	0.5341	0.1580	−0.4518	0.101*	
H7C	0.6427	0.2670	−0.3841	0.101*	

### Atomic displacement parameters (Å^2^)

**Table:**

	*U*^11^	*U*^22^	*U*^33^	*U*^12^	*U*^13^	*U*^23^
P1	0.0279 (2)	0.0352 (2)	0.0273 (2)	−0.00341 (13)	0.01246 (16)	−0.00360 (13)
O1	0.0347 (8)	0.0594 (11)	0.0381 (8)	−0.0165 (8)	0.0151 (6)	−0.0108 (7)
O2	0.0392 (8)	0.0322 (7)	0.0528 (8)	0.0013 (5)	0.0176 (6)	−0.0072 (6)
O3	0.0375 (8)	0.0562 (9)	0.0320 (7)	0.0005 (6)	0.0194 (6)	0.0036 (6)
O1'	0.045 (4)	0.034 (4)	0.044 (4)	−0.014 (3)	0.027 (4)	−0.011 (3)
O2'	0.027 (3)	0.062 (4)	0.039 (4)	0.006 (3)	0.019 (3)	−0.006 (3)
O3'	0.038 (4)	0.053 (4)	0.031 (3)	−0.004 (3)	0.004 (3)	0.005 (3)
O4	0.0373 (6)	0.0325 (5)	0.0290 (5)	−0.0056 (4)	0.0172 (4)	−0.0023 (4)
O5	0.0465 (7)	0.0676 (8)	0.0418 (7)	0.0217 (6)	0.0284 (6)	0.0121 (6)
N1	0.0413 (7)	0.0568 (8)	0.0342 (7)	0.0167 (6)	0.0214 (6)	0.0118 (6)
C1	0.0347 (8)	0.0538 (10)	0.0550 (11)	0.0079 (7)	0.0149 (7)	−0.0083 (8)
C2	0.0593 (13)	0.0810 (15)	0.0554 (12)	0.0133 (11)	0.0237 (10)	0.0319 (11)
C3	0.0318 (7)	0.0271 (6)	0.0287 (6)	−0.0031 (5)	0.0152 (5)	−0.0037 (5)
C4	0.0429 (9)	0.0498 (9)	0.0429 (9)	0.0172 (7)	0.0214 (7)	0.0102 (7)
C5	0.0287 (7)	0.0333 (7)	0.0305 (7)	0.0020 (5)	0.0156 (6)	−0.0014 (5)
C6	0.0318 (7)	0.0369 (7)	0.0298 (7)	0.0020 (5)	0.0147 (6)	0.0005 (5)
C7	0.0618 (13)	0.1063 (19)	0.0439 (10)	0.0368 (13)	0.0317 (10)	0.0331 (11)

### Geometric parameters (Å, °)

**Table:**

P1—O1	1.4472 (14)	C1—H1D	0.9800
P1—O1'	1.501 (7)	C1—H1E	0.9800
P1—O2'	1.536 (6)	C1—H1F	0.9800
P1—O2	1.5503 (14)	C2—H2A	0.9800
P1—O3	1.5536 (13)	C2—H2B	0.9800
P1—O4	1.5775 (11)	C2—H2C	0.9800
P1—O3'	1.580 (7)	C2—H2D	0.9800
O2—C1	1.439 (2)	C2—H2E	0.9800
O3—C2	1.446 (2)	C2—H2F	0.9800
O2'—C1	1.551 (7)	C3—C5	1.321 (2)
O3'—C2	1.382 (8)	C3—C4	1.479 (2)
O4—C3	1.4130 (16)	C4—H4A	0.9800
O5—C6	1.2250 (19)	C4—H4B	0.9800
N1—C6	1.340 (2)	C4—H4C	0.9800
N1—C7	1.442 (2)	C5—C6	1.487 (2)
N1—H1N	0.8800	C5—H5A	0.9500
C1—H1A	0.9800	C7—H7A	0.9800
C1—H1B	0.9800	C7—H7B	0.9800
C1—H1C	0.9800	C7—H7C	0.9800
			
O1—P1—O1'	37.3 (3)	H1B—C1—H1F	140.3
O1—P1—O2'	138.0 (3)	H1C—C1—H1F	109.3
O1'—P1—O2'	115.2 (4)	H1D—C1—H1F	109.5
O1—P1—O2	111.70 (10)	H1E—C1—H1F	109.5
O1'—P1—O2	75.8 (3)	O3'—C2—O3	54.0 (3)
O2'—P1—O2	47.1 (3)	O3'—C2—H2A	148.2
O1—P1—O3	117.47 (9)	O3—C2—H2A	109.5
O1'—P1—O3	139.1 (3)	O3'—C2—H2B	61.9
O2'—P1—O3	57.3 (3)	O3—C2—H2B	109.5
O2—P1—O3	103.86 (8)	H2A—C2—H2B	109.5
O1—P1—O4	113.96 (8)	O3'—C2—H2C	102.0
O1'—P1—O4	117.5 (3)	O3—C2—H2C	109.5
O2'—P1—O4	107.5 (3)	H2A—C2—H2C	109.5
O2—P1—O4	106.74 (7)	H2B—C2—H2C	109.5
O3—P1—O4	101.93 (7)	O3'—C2—H2D	109.5
O1—P1—O3'	73.0 (3)	O3—C2—H2D	148.9
O1'—P1—O3'	107.2 (4)	H2A—C2—H2D	69.9
O2'—P1—O3'	102.6 (4)	H2B—C2—H2D	47.5
O2—P1—O3'	141.6 (3)	H2C—C2—H2D	99.4
O3—P1—O3'	48.4 (3)	O3'—C2—H2E	109.5
O4—P1—O3'	105.2 (3)	O3—C2—H2E	101.3
C1—O2—P1	123.79 (13)	H2A—C2—H2E	100.0
C2—O3—P1	120.71 (13)	H2B—C2—H2E	126.0
P1—O2'—C1	117.4 (5)	H2C—C2—H2E	16.6
C2—O3'—P1	123.3 (5)	H2D—C2—H2E	109.5
C3—O4—P1	121.57 (9)	O3'—C2—H2F	109.5
C6—N1—C7	121.65 (15)	O3—C2—H2F	62.3
C6—N1—H1N	119.2	H2A—C2—H2F	47.2
C7—N1—H1N	119.2	H2B—C2—H2F	123.9
O2—C1—O2'	48.6 (3)	H2C—C2—H2F	126.0
O2—C1—H1A	109.5	H2D—C2—H2F	109.5
O2'—C1—H1A	139.2	H2E—C2—H2F	109.5
O2—C1—H1B	109.5	C5—C3—O4	117.41 (13)
O2'—C1—H1B	63.5	C5—C3—C4	130.16 (14)
H1A—C1—H1B	109.5	O4—C3—C4	112.32 (12)
O2—C1—H1C	109.5	C3—C4—H4A	109.5
O2'—C1—H1C	110.6	C3—C4—H4B	109.5
H1A—C1—H1C	109.5	H4A—C4—H4B	109.5
H1B—C1—H1C	109.5	C3—C4—H4C	109.5
O2—C1—H1D	141.2	H4A—C4—H4C	109.5
O2'—C1—H1D	109.5	H4B—C4—H4C	109.5
H1A—C1—H1D	63.8	C3—C5—C6	125.23 (13)
H1B—C1—H1D	49.0	C3—C5—H5A	117.4
H1C—C1—H1D	108.5	C6—C5—H5A	117.4
O2—C1—H1E	108.4	O5—C6—N1	122.15 (15)
O2'—C1—H1E	109.5	O5—C6—C5	124.98 (14)
H1A—C1—H1E	110.5	N1—C6—C5	112.87 (13)
H1B—C1—H1E	109.5	N1—C7—H7A	109.5
H1C—C1—H1E	1.2	N1—C7—H7B	109.5
H1D—C1—H1E	109.5	H7A—C7—H7B	109.5
O2—C1—H1F	64.3	N1—C7—H7C	109.5
O2'—C1—H1F	109.5	H7A—C7—H7C	109.5
H1A—C1—H1F	48.2	H7B—C7—H7C	109.5
			
O1—P1—O2—C1	−178.29 (15)	O2—P1—O3'—C2	84.0 (7)
O1'—P1—O2—C1	171.5 (3)	O3—P1—O3'—C2	31.1 (4)
O2'—P1—O2—C1	−42.1 (4)	O4—P1—O3'—C2	−61.4 (6)
O3—P1—O2—C1	−50.73 (17)	O1—P1—O4—C3	−38.76 (15)
O4—P1—O2—C1	56.53 (16)	O1'—P1—O4—C3	2.6 (4)
O3'—P1—O2—C1	−88.6 (5)	O2'—P1—O4—C3	134.5 (3)
O1—P1—O3—C2	−54.1 (2)	O2—P1—O4—C3	85.04 (12)
O1'—P1—O3—C2	−93.8 (5)	O3—P1—O4—C3	−166.34 (11)
O2'—P1—O3—C2	174.5 (4)	O3'—P1—O4—C3	−116.6 (3)
O2—P1—O3—C2	−178.02 (16)	P1—O2—C1—O2'	40.5 (4)
O4—P1—O3—C2	71.16 (17)	P1—O2'—C1—O2	−37.8 (3)
O3'—P1—O3—C2	−28.7 (4)	P1—O3'—C2—O3	−30.9 (4)
O1—P1—O2'—C1	109.6 (5)	P1—O3—C2—O3'	30.5 (4)
O1'—P1—O2'—C1	71.9 (6)	P1—O4—C3—C5	−85.84 (15)
O2—P1—O2'—C1	35.6 (3)	P1—O4—C3—C4	97.63 (14)
O3—P1—O2'—C1	−154.4 (7)	O4—C3—C5—C6	−175.23 (13)
O4—P1—O2'—C1	−61.2 (6)	C4—C3—C5—C6	0.6 (3)
O3'—P1—O2'—C1	−171.9 (5)	C7—N1—C6—O5	1.7 (3)
O1—P1—O3'—C2	−172.4 (7)	C7—N1—C6—C5	−177.96 (19)
O1'—P1—O3'—C2	172.7 (6)	C3—C5—C6—O5	3.9 (3)
O2'—P1—O3'—C2	51.0 (7)	C3—C5—C6—N1	−176.46 (15)

### Hydrogen-bond geometry (Å, °)

**Table:**

*D*—H···*A*	*D*—H	H···*A*	*D*···*A*	*D*—H···*A*
N1—H1N···O1^i^	0.88	2.03	2.902 (2)	169
C4—H4B···O2^ii^	0.98	2.43	3.319 (2)	151

Symmetry codes: (i) *x*−1/2, −*y*+1/2, *z*−1/2; (ii) −*x*+3/2, *y*−1/2, −*z*+1/2.
